# Problem of a Rare Anomalous Hepatic Artery During Whipple Procedure

**DOI:** 10.4103/1319-3767.61243

**Published:** 2010-04

**Authors:** Aswini K. Pujahari

**Affiliations:** Sr. Adv. GI Surgery, Department of Surgery, Command Hospital (AF) Bangalore - 560 007, Karnataka, India E-mail: akpuja@rediffmail.com

Sir,

Anatomical variation of the Hepatic Artery (HA) is seen in 20.4% of liver donors. The common variations include a replaced or an accessory right HA originating from the superior mesenteric artery (6.67%) and a replaced or an accessory left HA originating from the left gastric artery (6.41%).[1]

**Figure 1:**
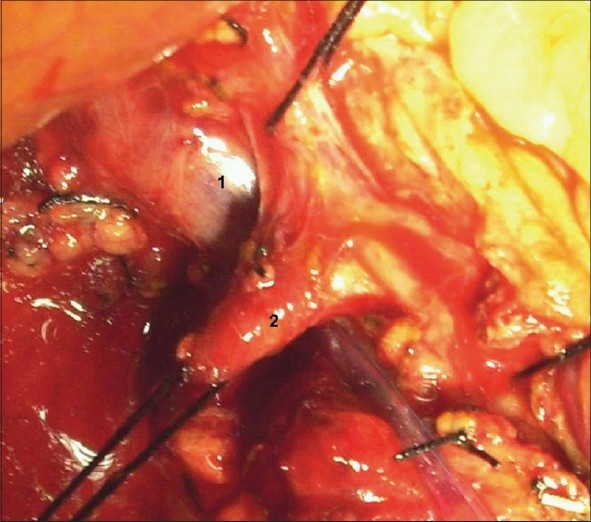
Hepatic artery before gastroduodenal disconnection. (1) Portal vein, (2) Abrrant hepatic artery

**Figure 2:**
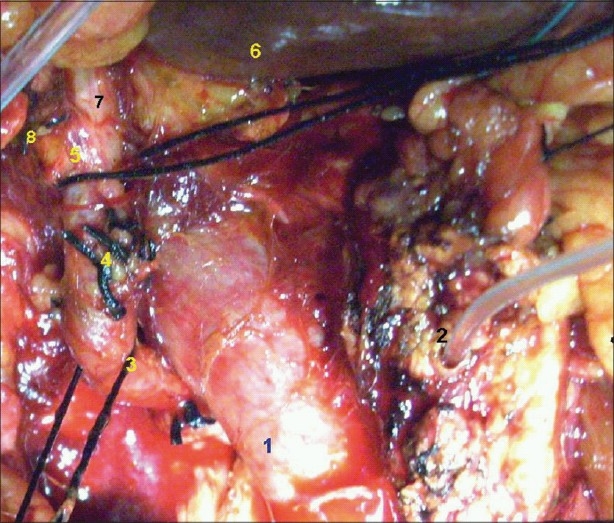
Hepatic artery after division of the gastroduodenal artery. (1) Portal vein, (2) Main pancreatic duct with tube inside, (3) Aberrant hepatic artery, (4) Ligated gastro-duodenal artery, (5) Common hepatic artery, (6) Liver, (7) Left hepatic artery 8 Right hepatic artery with ligated cystic artery

A 46-year-old male patient presented with painless and progressive jaundice of 1 month's duration, with severe generalized itching and white stools. Clinically, he was deeply jaundiced, with serum bilirubin of 26mg% and elevated alkaline phosphatase. The whole biliary tree was seen to be dilated on Ultrasonography (USG). Side-viewing endoscopic biopsy from an ulcer at the ampulla was reported as adenocarcinoma. There was no metastasis on evaluation. While doing the classical Whipple procedure the HA was dissected free in the normal location in front of and left of the portal vein. While dissecting toward the gastroduodenal artery, a much bigger vessel was seen crossing the portal vein anteriorly. The main artery was seen to the right of the portal vein, crossing in front of the portal vein at the superior border of the pancreatic neck and dividing into the gastroduodenal and the common HA, which further divided into the right and left HA [Figures 1 and 2]. There was no HA arising from the celiac trunk. As the artery lay in front of the portal vein, along the line of the pancreatic neck transection, the chance of injury was high. The main trunk and the artery beyond the gastroduodenal were protected and an uneventful Whipple procedure was done.

The arterial system of the liver in humans presents wide variability and knowledge of the different variations is important when operating in this region.[2] In a cadaveric dissection, a similar anatomical variation has been reported, with a gastrosplenic and hepatomesenteric trunk, and with the HA having a similar course to that found in our case[3]; we, of course, could not dissect till the origin of the artery in our live patient. In another cadaveric dissection, two anomalous HAs were described; one of these was similar to the HA seen in the present case, which can be classified as type VI of Adachi's classification and type IV of Morita's classification.[4] Ours is the first report of such an anomaly in a live jaundiced patient. Such cases require careful dissection and ligature of the gastroduodenal artery, with care being taken to preserve the common hepatic trunk.
